# High Maternal Triglyceride Levels Mediate the Association between Pre-Pregnancy Overweight/Obesity and Macrosomia among Singleton Term Non-Diabetic Pregnancies: A Prospective Cohort Study in Central China

**DOI:** 10.3390/nu14102075

**Published:** 2022-05-16

**Authors:** Xinli Song, Letao Chen, Senmao Zhang, Yiping Liu, Jianhui Wei, Mengting Sun, Jing Shu, Tingting Wang, Jiabi Qin

**Affiliations:** 1 Department of Epidemiology and Health Statistics, Xiangya School of Public Health, Central South University, Changsha 410078, China; xinlisong@foxmail.com (X.S.); chenletao93@163.com (L.C.); zsmhncs@163.com (S.Z.); 18843113354@163.com (Y.L.); weijihi@163.com (J.W.); sunmtbeloved@163.com (M.S.); sj1234511@163.com (J.S.); 2 National Health Committee Key Laboratory of Birth Defect for Research and Prevention, Hunan Provincial Maternal and Child Health Care Hospital, Changsha 410028, China; 3 Guangdong Cardiovascular Institute, Guangdong Provincial People’s Hospital, Guangdong Academy of Medical Sciences, Guangzhou 510080, China; 4 Hunan Provincial Key Laboratory of Clinical Epidemiology, Changsha 410078, China

**Keywords:** overweight, obesity, macrosomia, triglyceride, gestational diabetes mellitus, mediation analysis

## Abstract

This study aimed at examining the risk of macrosomia, in relation to maternal pre-pregnancy overweight/obesity mediated via high maternal triglyceride (mTG) levels. In this prospective study, 24,730 singleton term non-diabetic pregnancies were finally included. Serum mTG levels were measured using fasting blood samples that were collected after 28 weeks of gestation. High mTG levels were defined as values ≥ the 90th percentile. The outcome of interest was macrosomia (≥4000 g). Log-binomial regression was used to assess the mediation path between overweight/obesity, high mTG levels, and macrosomia. The mediation analysis found a total effect of overweight on macrosomia of 0.006 (95% CI, 0.001–0.010), including a direct effect of 0.005 (95% CI, 0.001, 0.009) and indirect effect of 0.001 (95% CI, 0.000–0.001), with an estimated proportion of 11.1% mediated by high mTG levels. Additionally, we also found a total effect of obesity on macrosomia of 0.026 (95% CI, 0.018–0.036), including a direct effect of 0.025 (95% CI, 0.017–0.036) and indirect effect of 0.001 (95% CI, 0.000–0.001), with an estimated proportion of 3.8% mediated by high mTG levels. In conclusion, non-diabetic women with overweight or obesity had an increased risk of macrosomia, and this positive association was partly mediated by high mTG levels.

## 1. Introduction

Macrosomia, a term applied to denote excessive fetal growth, is defined as a birth weight equal to or beyond 4000 g, independent of gestational age and fetal sex [1]. There is growing interest regarding the impact of macrosomia on both mother and child. Macrosomia is associated with an increased risk of postpartum hemorrhage and obstetric anal sphincter injuries for mothers, as well as with labor abnormalities, hypoxia, shoulder dystocia, and nerve injuries for newborns [2,3]. In addition, it also predisposes children to a series of adverse health outcomes that will persist throughout their lifespan, such as obesity, metabolic disorders, cardiovascular complications, and non-alcoholic fatty liver disease [4,5]. Macrosomia is commonly encountered in obstetric practice, with a prevalence of approximately 4–10% of Chinese newborns, varying by region [6,7].

The body mass index (BMI) in Chinese women of reproductive age (18–44 years) has been rising over the past decade [8], and overweight and obesity are positively correlated with gestational diabetes mellitus (GDM) and dyslipidemia [9], posing a key clinical and public health concern. GDM is regarded to be the most significant pregnancy complication causing macrosomia, with approximately 15–45% of macrosomic babies born to mothers with GDM [10,11]. The Pedersen’s hypothesis suggests that fetal overgrowth is associated with increased glucose consumption and fetal hyperinsulinemia, due to maternal hyperglycemia [12]. Excess nutrients being shunted to the fetus is known to increase the risk of fetal overgrowth [13], though in newborns born to women without GDM [14]. Maternal lipids are also important substrates for optimal fetal development, but they are less appreciated. The Hyperglycemia and Adverse Pregnancy Outcome study provided evidence that maternal glucose affects neonatal size in a sustained manner; maternal lipid levels were not mentioned [15]. The major lipid contributor, serum triglycerides, increase physiologically as gestation progresses, owing to increases in estrogen, progesterone, and lactogen, to maintain pregnancy and fetal growth [16]. It has been conclusively demonstrated that elevated maternal triglyceride (mTG) can carry an increased risk of giving birth to macrosomia [17], even in obese mothers with normal glucose tolerance [18], implying that the role of mTG is as important as the well-established role of glucose in influencing birth weight [19]. The hydrolysates of mTG, such as free fatty acids, may be transported by placental trophoblasts via specific fatty acid binding/transport proteins [20]. As a result, the mechanisms underlying the role of mTG in macrosomia may incorporate metabolic adaptations in response to increased transport of specific fatty acids to the placenta and fetus [21].

High mTG levels are a known consequence of overweight/obesity and risk factor for macrosomia, indicating that high mTG levels may be on this causal pathway. Actually, Lu and colleagues have reported that mTG has a significant mediating effect on the relationship between pre-pregnancy BMI and macrosomia [22]. Lipid metabolism in normal glucose-tolerant pregnancies differs from that in GDM pregnancies, where a rise of insulin promotes the maternal fat depots and increases the subsequent hypertriglyceridemia, due to insulin resistance and estrogen-like effects [23]. Hence, it is necessary to explore the role of elevated mTG levels on the risk of macrosomia in non-diabetic women, in order to corroborate and extend the observations of Lu and colleagues. The current study puts forward an assumption that high mTG levels may act as a mediator in the association between overweight/obesity and macrosomia in non-diabetic women. With this context in mind, the purpose of this prospective study was to determine the degree to which the association of pre-pregnancy overweight or obesity with the risk of macrosomia in singleton full-term, non-diabetic pregnancies is mediated by high mTG levels in late pregnancy, thus contributing a piece to a complex puzzle.

## 2. Materials and Methods

### 2.1. Study Population and Data Collection

**Figure A1:**
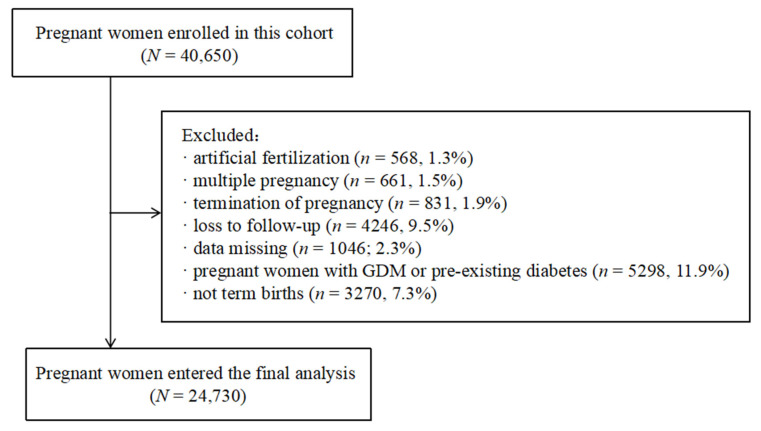
Flow chart presenting the participant’s recruitment.

This cohort recruitment began on 13 March 2013 and was carried out in a large maternal and child health center that served patients from all over Hunan Province in Central China. We included first-trimester pregnant women (≥18 years) who planned to receive continuous prenatal care (until delivery) at the study hospital. The last menstrual period was used as the dating method, in order to estimate the gestational age in weeks, which was then tracked by ultrasound at the first antenatal visit. As of December 2020, a total of 40,650 pregnant women who met the inclusion criteria were recruited in this cohort. Owing to missing data, loss to follow-up, and some participants meeting the exclusion criteria, 24,730 pregnant women without GDM who delivered singleton term babies (37–41 completed weeks gestation) were finally included in the analysis, and the detailed reasons were as follows: (i) artificial fertilization (*n* = 568, 1.3%); (ii) multiple pregnancy (*n* = 661, 1.5%); (iii) termination of pregnancy (*n* = 831, 1.9%); (iv) loss to follow-up (*n* = 4246, 9.5%); (v) data missing (*n* = 1046; 2.3%); (vi) pregnant women with GDM or pre-existing diabetes (*n* = 5298, 11.9%); and (vii) not term births (*n* = 3270, 7.3%) (Figure A1). This study was conducted in accordance with the principles of the Declaration of Helsinki and granted by the Ethics Committee for Clinical Research of Xiangya School of Public Health of Central South University (no. XYGW-2018-36). Before data collection, all participants agreed to provide written informed consent. In addition, this study was registered in the Chinese Clinical Trial Registry Center (registration number: ChiCTR1800016635).

At registration for pregnancy, our specially trained research staff carried out a face-to-face interview with each enrolled woman, using the study-specific questionnaire to collect maternal data, in detail, as follows: age at pregnancy onset (<25, 25–29, 30–34, or ≥35), education (high school or less, some college, or bachelor’s or higher), parity (primipara or multipara), smoking (yes or no), and drinking (yes or no). Specifically, smoking was defined as consuming one or more cigarettes per day for at least three months before or during gestation. The definition of drinking was consuming alcohol one or more times a week before or during gestation. Furthermore, maternal height and weight were measured at the initial of recruitment to obtain each eligible pregnant woman’ body mass index (BMI), the exposure of interest in this study, which was classified using Chinese adult criteria: underweight (<18.5 kg/m^2^), normal weight (18.5–23.9 kg/m^2^), overweight (24.0–27.9 kg/m^2^), and obesity (≥28.0 kg/m^2^) [24]. We tracked each participant’s maternal weight in the weeks leading up to delivery and calculated the difference between it and the initial measured weight as gestational weight gain (<10, 10–20, or ≥20 kg). The clinical records for pregnant women (e.g., the diagnosis of GDM and gestational hypertension) and delivery (e.g., infant sex and birth weight) were retrieved from the database of the hospital’s electronic medical records.

High mTG levels, as a potential mediator, are a known effect of maternal obesity and a risk factor for fetal macrosomia. Fasting blood sampling was carried out among all participants after 28 weeks of gestation to measure serum triglyceride levels, and the exact gestational weeks of the test were recorded. A commercial enzymatic assay (Roche Diagnostics, Mannheim, Germany) and Cobas c702 analyzer were used to determine serum triglyceride levels, with the interassay coefficient of variation < 2.3%. Because mTG levels increased with gestational age, they were corrected for gestational age at the time of blood collection. For comparison, mTG levels were divided using the 90th percentile as the cut-off point. As a result, “low mTG levels” meant values below the 90th percentile, and “high mTG levels” meant values equal to or above the 90th percentile [25,26]. Macrosomia, as the outcome, was denoted as a birth weight of 4000 g or more, regardless of gestational age and infant sex [1]. Sensitivity analysis was performed by using the 80th percentile as the cut-off point for dividing mTG levels to test for consistency.

### 2.2. Statistical Analyses

**Figure 1:**
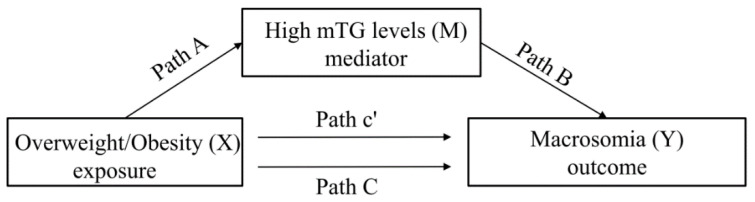
The illustration of the total (Path C), direct (Path c’), and indirect (Path A and B) effects of the association between the exposure (X) and outcome (Y) via the mediator (M). Abbreviation: mTG = maternal triglyceride.

In this study, including 24,730 singleton full-term pregnancies without GDM, the baseline characteristics of pregnant women and infants were described as a percentage, according to high mTG levels and macrosomia births. The prevalence rates and their 95% confidence intervals (95% CIs) of high mTG levels and fetal macrosomia were estimated across maternal pre-pregnancy BMI categories. We hypothesized that high mTG levels may act as a mediator in the association between pre-pregnancy overweight/obesity and risk of fetal macrosomia. The mediation analysis decomposed the total effect of an association into direct and indirect effects, i.e., the effect of causal pathway via a specific mediator. This mediating effect existed on the premise that the association between overweight/obesity and high mTG levels (Path A), high mTG levels and newborn macrosomia (Path B), and overweight/obesity and newborn macrosomia (Path C) were all statistically significant (Figure 1). Hence, we examined these associations using log-binomial (log-linear) regression models, adjusted for pre-pregnancy overweight/obesity (only for Path B), age at pregnancy onset, education, smoke, drink, parity, infant sex, gestational weight gain, gestational hypertension, and mTG levels (only for Path C), and reported their effect estimates as relative risk ratios (RRs) and the corresponding 95% CIs. Subsequently, using the mediation package in R software, the mediation analysis was performed to estimate the total, indirect, and direct effects, in addition to a quasi-Bayesian Monte Carlo method with 5000 simulations, on the basis that a normal approximation was applied to achieve uncertainty estimation [27]. The mediated proportion, i.e., the value of indirect effect divided by the total effect, was to evaluate how much the association of pre-pregnancy overweight or obesity with the risk of macrosomia was mediated via high mTG levels, and this was calculated using R software. A two-tailed *p* value < 0.05 was considered to indicate statistical significance. All statistical analyses were carried out using R version 3.6.2 (R Foundation for Statistical Computing, Vienna, Austria).

## 3. Result

### 3.1. Baseline Characteristics of the Participants

This study included 24,730 participants in the final analyses, and the distribution of characteristics of pregnant women and neonates is provided in Table 1. Overall, the prevalence of high mTG levels and macrosomia births were approximately 10.1% and 3.9%, respectively. Out of the total participants, overweight and obese women accounted for 11.4% and 2.2%, respectively. The majority of women were between the ages of 25 and 34, with 36.8% aged 25 to 29 and 37.4% aged 30 to 34. In our study sample, approximately half of the women had a college degree (51.3%), and nearly seven out of 10 women (74.2%) had a normal gestational weight gain (kg), ranging from 10 to 20. In addition, only 1.0% of the participants had experience with smoking, and 1.5% was exposed to alcohol. Furthermore, we found that the distribution of maternal and infant’ characteristics in the high mTG levels and fetal macrosomia groups was basically consistent with the counterparts of the total births.

### 3.2. Prevalence of High mTG Levels and Fetal Macrosomia across Pre-Pregnancy BMI Categories

Table 2 showed the prevalence of high mTG levels and fetal macrosomia according to maternal pre-pregnancy BMI status. Obese women had the highest prevalence of high mTG levels and macrosomia (14.4% and 11.6%, respectively), which was higher than that of overweight women (13.1% and 5.0%, respectively) and women with normal BMI (9.9% and 3.7%, respectively).

### 3.3. Testing for Significance of Path A, B, and C

Path A was used as a mediator model to estimate the effects of overweight/obesity on high mTG levels, and positive and significant associations were observed after potential confounders were adjusted (overweight RR = 1.35, 95% CI, 1.20–1.53; obesity RR = 1.48, 95% CI, 1.15–1.91) (Table 3). Path B, as an outcome model, was applied to calculate the estimated effects of high mTG levels on newborn macrosomia; after the selected confounding factors were adjusted, positive and significant associations were found (overweight RR = 2.26, 95% CI, 1.89–2.72; obesity RR = 1.86, 95% CI, 1.52–2.28). In addition, the total effects of overweight/obesity on fetal macrosomia were estimated in the Path C model, and positive and significant associations were observed after adjustment for potential confounders (overweight RR = 1.45, 95% CI, 1.20–1.76; obesity RR = 4.26, 95% CI, 3.20–5.68).

### 3.4. Mediation Analysis

The estimates of the total, direct, and indirect effects, in association with maternal overweight/obesity on macrosomic newborns with high mTG levels in late pregnancy as a mediator, were statistically significant and larger than 1 (Table 4). A direct effect greater than 1 indicated that overweight/obese mothers exhibited a significantly higher risk of delivering a macrosomic baby without the effect of high mTG levels, while an indirect effect greater than 1 suggested that high mTG levels increased the risk of macrosomic babies born to overweight/obese mothers. Specifically, the mediation analysis found a total effect of overweight on macrosomia of 0.006 (95% CI, 0.001–0.010; *p* < 0.01), including a direct effect of 0.005 (95% CI, 0.001, 0.009; *p* < 0.05) and indirect effect of 0.001 (95% CI, 0.000–0.001; *p* < 0.001), with an estimated proportion of 11.1% mediated by high mTG levels. Additionally, we also found a total effect of pre-pregnancy obesity on macrosomia of 0.026 (95% CI, 0.018–0.036; *p* < 0.001), including a direct effect of 0.025 (95% CI, 0.017–0.036; *p* < 0.001) and indirect effect of 0.001 (95% CI, 0.000–0.001); *p* < 0.05). Though the indirect effect in the obesity group was similar to that in the overweight group, a lower proportion estimate of 3.8% mediated by high mTG levels was observed among obese women.

Furthermore, to test the consistency, we redefined “high mTG level” and “low mTG level”, with the 80th percentile as the cut-off point, and repeated the mediation analysis with it as a mediator. As shown in Table A1, the results of the total, direct, and indirect effects observed in the overweight and obese groups were extremely similar.

## 4. Discussion

Maternal pre-pregnancy overweight and obesity are positively correlated with an increased risk of a variety of gestational complications, including dyslipidemia. According to growing evidence, maternal factors related to fetal overgrowth are not only limited to maternal glucose availability; maternal lipids, such as triglyceride, are also important contributors for birth weight, increasing the risk of fetal macrosomia. Considering the complex effect of GDM on lipids changes during pregnancy [28], this large, hospital-based cohort study focused on non-diabetic women and aimed to understand the role of elevated mTG levels as a mediator, in association with maternal pre-pregnancy overweight/obesity and macrosomia in singleton full-term pregnancies.

Gestational normal metabolism is coordinated by placental hormones and accompanied by a physiologic rise in serum insulin, glucose, and lipids, such as triglyceride. Maternal fat accumulation culminates in the second trimester and declines in the third trimester, resulting in the serum mTG levels being in an elevated state as gestation advances to maintain stable fuel dispatch to the fetus [29]. Triglycerides are important contributors to fetal fat accretion and excess growth, but they are less appreciated than the well-recognized glucose. Our study found that elevated fasting serum mTG levels determined in late pregnancy exhibited a significant correlation with the risk of macrosomia in non-diabetic women with overweight or obesity. A recent meta-analysis, including 42 prospective and retrospective studies, showed a relatively comprehensive summary of the associations between first-, second-, or third-trimester mTG levels (fasting, postprandial, or random) and the risk of offspring macrosomia in women of different races/ethnicities [30], with the majority of studies yielding positive and significant associations. In addition, Cianni and colleagues observed that fasting serum mTG determined in the third trimester of pregnancy was independently associated with newborn weight in women with normal glucose tolerance [31], which was basically consistent with our findings. Kitajima and colleagues found fasting triglyceride levels at 24–32 weeks associated positively with neonatal birth weight at term in women with normal glucose tolerance [32], which also provided evidence for our observations. Furthermore, Kulkarni and colleagues found that serum total cholesterol and triglycerides levels at 18 and 28 weeks of gestation and serum glucose levels at only 28 weeks of gestation were positively related to birth weight in a group of Indian rural women who were malnourished and had a low prevalence of diabetes [33]. These data seemed to suggest that, on the one hand, the role of maternal lipids was as important as the well-established effect of glucose on birth weight, and, on the other hand, the effect of mTG levels on birth weight emerged as early as the first trimester of pregnancy. What’s more, it was demonstrated that, in mothers with obesity, who also had normal glucose tolerance, mTG may be a more potent promoter of fetal overgrowth than glucose [19]. Harmon and colleagues conducted a clinical trial of controlled diets and found that, in women with normal glucose tolerance, infant percentage body fat was associated with maternal BMI, glucose, and insulin, but triglyceride was the strongest predictor [19].

Previous efforts, aimed predominantly at glycemic control to reduce LGA births or fetal macrosomia, were unsatisfactory as a whole, with diet, metformin, or myoinositol applied in different randomized controlled trials [20]. In fact, overweight/obese women without GDM were common, and they were also at high risk for macrosomia/LGA deliveries, but more or less neglected, compared to those with GDM. No drugs were approved for use during their gestation, so appropriate dietary and lifestyle interventions that are targeted at lowering mTG levels to normalize birth weight may be critical. Few studies reported inconsistent associations between fat-modified dietary interventions during pregnancy and mTG levels, and they did not find a significant effect of these interventions on birth weight [34,35]. Additionally, the beneficial effects of omega-3 fatty acids (long-chain polyunsaturated fatty acids) on altering of maternal lipids and improving perinatal outcomes were of great attention. A recent systematic review and meta-analysis was conducted, based on pregnant women, and observed that no significant effects of omega-3 fatty acids supplementation on fasting glucose, insulin, insulin resistance, total cholesterol, and triglycerides were found [36]. In addition, it was shown that the maternal intake of omega-3 fatty acids during gestation caused a mildly prolonged gestation and slightly higher birth weights [37]. However, the discrepancies in birth weights between the intervention and control groups disappeared when controlling the gestational age as a confounder [38]. Furthermore, in a meta-analysis including 31 randomised trials (5278 newborns), the effect of lifestyle interventions during pregnancy on fetal weight was evaluated, and a small, but non-significant, reduction in birth weight was found, compared to the control group [39]. Overall, numerous randomized controlled trials have endeavored to normalize fetal birth weight, with the goal of lowering mTG or glucose levels by means of diet, physical activity, or mixed interventions, but reaped very limited benefits.

Our findings showed that high mTG levels mediated the association between maternal overweight or obesity and fetal macrosomia in non-diabetic women, with the mediated proportion being approximately 11.1% and 1.5% in overweight and obese women, respectively. From overweight to obesity, the risk of women suffering high mTG levels in late pregnancy, as well as the corresponding mediating effects on offspring macrosomia, were not increased, but elevated direct effects were observed. This interesting finding seemed to represent the “saturation” of the effect of pre-pregnancy BMI from overweight to obesity on mTG levels, which could be explained by previous observations. Overweight/obese women commenced gestation with higher TG levels than normal weight women, but then they reached a similar maximum [40]. In addition, Farias and colleagues recorded lipids changes throughout pregnancy, according to pre-pregnancy BMI, and found lower rates of change in the serum TG levels of overweight/obese women, compared to normal-weight controls [41].

Lu and colleagues considered BMI to be a continuous variable and reported that the proportion of high mTG levels mediating the association between pre-pregnancy BMI and macrosomia was approximately 15.7% in all pregnancies [22]. Obviously, their observations were not comparable to ours, due to differences in the inclusion and exclusion criteria of participants and type of variables, but both suggested a considerable direct role of obesity in microsomia, as well as other, as-yet unknown, mediating roles in the association between maternal obesity and infant birth weight. Fetuses born to overweight women were consistently above average in all fetal body composition parameters assessed [42], indicating that the direct role of overweight/obesity in fetal growth was early and significant. Furthermore, gestational metabolism possessed complex alterations in glycolipid metabolism that were regulated by the environment and genes [43,44]; metabolic factors, such as interleukin-17 [45], leptin [46], adiponectin [47], C-reactive protein [45], and so on, were correlated with newborn birth weight, though most mechanisms remained unknown. There was growing recognition of the importance of early excess nutrient delivery in affecting fetal overgrowth, due to overweight or obesity at the onset of pregnancy [48]. The evolving epidemic of overweight and obesity among the adult population in China has been demonstrated in the last decade, due to rapid urbanization and modernization [8]. To date, there is still an urgent need for safe, easy-to-apply, and effective prevention strategies to help overweight/obese women preparing for pregnancy to manage their weight, in order to prevent adverse outcomes, including macrosomia.

This study had several limitations that need to be addressed. First, all participants in this study were from an urban area; replication efforts, regarding our study findings, are needed in other pregnant populations. Second, the fatty acid profile of maternal and offspring was not tested, which may help explain the proposed association between high mTG levels and fetal macrosomia. Third, there were many other significant metabolic factors associated with mTG and macrosomia, such as interleukin-17, leptin, and adiponectin, as well as other key issues caused by maternal overweight/obesity and the imbalance of lipid metabolism, such as non-alcoholic fatty liver disease in children later in life, which requires more in-depth studies.

## 5. Conclusions

This study found that non-diabetic women who were overweight/obese prior to pregnancy had an increased risk of fetal macrosomia, and this positive association was mediated, in part, by high maternal TG levels, emphasizing the important role of obesity and potential role of high TG levels in fetal overgrowth. Concerns about fetal overgrowth should be included in weight-control intervention strategies targeting overweight or obese women commencing their pregnancies.

## Figures and Tables

**Table 1:** Distribution of maternal and infant characteristics in the study sample of singleton births (*N* = 24,730), according to high mTG and macrosomia births.

Maternal and Infant Characteristics	Total Births *n* (%)	High mTG *n* (%)	Macrosomia *n* (%)
	*N* = 24,730	2487 (10.1)	959 (3.9)
Pre-pregnancy BMI (kg/m^2^)			
Underweight (<18.5)	3781 (15.3)	301 (12.1)	111 (11.6)
Normal (18.5–23.9)	17,593 (71.1)	1739 (69.9)	646 (67.4)
Overweight (24.0–27.9)	2822 (11.4)	370 (14.9)	140 (14.6)
Obese (≥28.0)	534 (2.2)	77 (3.1)	62 (6.5)
Age at pregnancy onset (year)			
<25	1351 (5.5)	150 (6.0)	57 (5.9)
25–29	9103 (36.8)	732 (29.4)	367 (38.3)
30–34	9248 (37.4)	955 (38.4)	359 (37.4)
≥35	5028 (20.3)	650 (26.1)	176 (18.4)
Education			
High school or less	8625 (34.9)	928 (52.0)	321 (33.5)
Some college	12,687 (51.3)	1293 (37.3)	530 (55.3)
Bachelor’s or higher	3418 (13.8)	266 (10.7)	108 (11.3)
Smoke			
No	24,476 (99.0)	2459 (98.9)	946 (98.6)
Yes	254 (1.0)	28 (1.1)	13 (1.4)
Drink			
No	24,353 (98.5)	2440 (98.1)	943(98.3)
Yes	377 (1.5)	47 (1.9)	16(1.7)
Parity			
Primipara	12,088 (48.9)	1157 (46.5)	484 (50.5)
Multipara	12,642 (51.1)	1330 (53.5)	475 (49.5)
Infant sex			
Male	13,184 (53.3)	1318 (53.0)	645 (67.3)
Female	11,546 (46.7)	1169 (47.0)	314 (32.7)
Gestational weight gain (kg)			
<10	18,353 (13.6)	1798 (13.4)	684 (6.6)
10–20	3373 (74.2)	333 (72.3)	63 (71.3)
≥20	3004 (12.1)	356 (14.3)	212 (22.1)
Gestational hypertension			
No	23,950 (96.8)	2414 (97.1)	935 (97.5)
Yes	780 (3.2)	73 (2.9)	24 (2.5)

Abbreviations: mTG = maternal triglyceride; BMI = body mass index.

**Table 2:** The prevalence of high mTG levels and fetal macrosomia across maternal pre-pregnancy BMI categories.

Category	High mTG % (95% CI)	Macrosomia % (95% CI)
Normal (18.5–23.9)	9.9 (9.4–10.3)	3.7 (3.4–3.9)
Overweight (24.0–27.9)	13.1 (11.9–14.4)	5.0 (4.2–5.8)
Obese (≥28.0)	14.4 (11.4–17.4)	11.6 (8.9–14.3)

Abbreviations: mTG = maternal triglyceride; 95% CI = 95% confidence interval.

**Table 3:** Testing for significance of Path A, B, and C.

Category	Path A aRR (95%CI) ^a^	Path B aRR (95%CI) ^b^	Path C aRR (95%CI) ^c^
Overweight	1.35 (1.20–1.53)	2.26 (1.89–2.72)	1.45 (1.20–1.76)
Obese	1.48 (1.15–1.91)	1.86 (1.52–2.28)	4.26 (3.20–5.68)

Note: Path A (mediator model): the effect of pre-pregnancy overweight/obesity on high mTG levels; Path B (outcome model): the effect of high mTG levels on newborn macrosomia; Path C: the total effect of pre-pregnancy overweight/obesity on newborn macrosomia. Abbreviation: mTG = maternal triglyceride; 95% CI = 95% confidence interval; RRs, relative risk ratios. ^a^ Adjusted for age at pregnancy onset, education, smoke, drink, parity, infant sex, gestational weight gain, and gestational hypertension. ^b^ Adjusted for pre-pregnancy overweight/obesity, age at pregnancy onset, education, smoke, drink, parity, infant sex, gestational weight gain, and gestational hypertension. ^c^ Adjusted forage at pregnancy onset, education, smoke, drink, parity, infant sex, gestational weight gain, gestational hypertension, and mTG levels.

**Table 4:** Mediation effects of high mTG levels on the association between pre-pregnancy overweight/obesity and fetal macrosomia.

Category	Total Effect (95% CI)	Direct Effect (95% CI)	Indirect Effect (95% CI)	Mediated Proportion, %
Overweight	0.006 (0.001–0.010) **	0.005 (0.001–0.009) *	0.001 (0.000–0.001) ***	11.1
Obese	0.026 (0.018–0.036) ***	0.025 (0.017–0.036) ***	0.001 (0.000–0.001) *	3.8

Note: Adjusted for age at pregnancy onset, education, smoke, drink, parity, infant sex, gestational weight gain, and gestational hypertension; * *p* < 0.05, ** *p* < 0.01, *** *p* < 0.001. Abbreviations: mTG = maternal triglyceride; 95% CI = 95% confidence interval.

## Data Availability

The data presented in this study are available on request from the corresponding author.

## Appendix A

**Table A1:** Mediation effects of high mTG levels on the association between pre-pregnancy overweight/obesity and fetal macrosomia, when mTG levels were divided using the 80th percentile as the cut-off point.

Category	Total Effect (95% CI)	Direct Effect (95% CI)	Indirect Effect (95% CI)	Mediated Proportion, %
Overweight	0.006 (0.001–0.010) **	0.005 (0.001–0.009) *	0.001 (0.000–0.001) ***	11.1
Obese	0.026 (0.018–0.036) ***	0.025 (0.018–0.036) ***	0.001 (0.000–0.001) *	3.8

Note: Adjusted for age at pregnancy onset, education, smoke, drink, parity, infant sex, gestational weight gain, and gestational hypertension; * *p* < 0.05, ** *p* < 0.01, *** *p* < 0.001. Abbreviations: mTG = maternal triglyceride; 95% CI = 95% confidence interval.
